# Effects of aviary opening times and dietary crude fiber contents on integument and keel bone condition in brown layer pullets

**DOI:** 10.1016/j.psj.2026.107213

**Published:** 2026-06-01

**Authors:** R. Schreiter, L. Fitz, J. Krieg, P. Schulte-Huxel, A. Schröder, J. Slama, P. Hofmann, H. Louton

**Affiliations:** a Martin Luther University Halle-Wittenberg, Theodor-Lieser-Straße 11, 06120 Halle, Germany; b Bavarian State Research Center for Agriculture, Kitzingen, Germany; c Chamber of Agriculture of North Rhine-Westphalia, Bad Sassendorf, Germany; d University of Rostock, Rostock, Germany; e Ludwig Maximilian University of Munich, Munich, Germany

**Keywords:** Pullets, Plumage condition, Dietary fiber, Aviary management

## Abstract

Plumage damage (PD) caused by severe feather pecking, skin injuries (SI) caused by cannibalism, and keel bone alterations present major challenges for animal welfare, performance and profitability in commercial pullet rearing and subsequent laying hen management. Preventive measures should already be implemented during rearing, but prospective longitudinal studies during the rearing period investigating how dietary fiber contents and aviary management influence the integument and keel bone condition of pullets are scarce.

Therefore, the aim of this study was to investigate the influence of the fiber content in pullet feeds and the timing of aviary opening on integument characteristics and keel bone deviations in pullets of a brown laying strain. A total of 3,872 non-beak-trimmed pullets (Lohmann Brown Classic) were kept in an aviary system with 16 compartments in a 2 × 2 factorial experimental design: Feed with a fiber content typically used under practical rearing conditions (crude fiber: 4.9% in grower, 5.8% in developer) vs. moderate elevated fiber feed (crude fiber: 6.8% in grower, 7.8% in developer) and early (day 14) vs. late (day 28) opening of the aviary levels. In weeks 4, 8, 12 and 16, PD to the back and belly, wing feather loss and SI were scored and the keel bone was palpated.

Binary logistic regression models showed significant associations between the dietary fiber content and the time of opening the aviary with PD (P ≤ 0.012) and SI (P ≤ 0.004), but not with wing feather loss (P ≥ 0.685) and keel bone deviations (P ≥ 0.148). Keel bone deviations were detected in 5.4% of birds at week 16. The back/belly plumage and skin showed more damage when the aviary was opened later and the fiber content was lower. For skin injuries, there was a significant interaction between feeding and the time of opening the aviary (P = 0.009), as the positive effect of fiber intake was only observed when the aviary was opened at day 28.

These results highlight the potential of targeted feeding and environmental strategies during rearing to promote integument health and reduce risk factors for PD and SI.

## Introduction

Feather pecking and cannibalism are major challenges for the welfare of laying hens in the rearing and laying phases, as they lead to plumage damage (**PD**), skin injuries (**SI**), and increased mortality, especially in flocks with intact beaks (Rodenburg et al., 2013; Nicol et al., 2013; Spindler et al., 2016). Therefore, feather and skin condition are widely recognized as important welfare indicators in pullets and young layers (Schreiter et al., 2022). Both feather pecking and cannibalism are non-aggressive behaviours (Savory, 1995) and have multifactorial causes, including genetics, feeding, housing and management (van Krimpen et al., 2005; Kjaer and Bessei, 2013; Preisinger, 2021). A key factor in preventing behavioural disorders within housing systems is access to litter and manipulable enrichment like alfalfa bales, pecking stones, straw or grain additions to litter (Janczak and Riber, 2015; van Staaveren et al., 2021). The provision of enrichment materials during the rearing period can also reduce feather pecking in the subsequent laying period (Schreiter et al., 2020).

In north-western Europe, multi-tier aviary systems are the dominant housing system in commercial layer production, and rearing systems are increasingly adapted to this structure to support a smooth transition to the laying environment (Heerkens et al., 2016; Lohmann Breeders, 2021). European guidelines (European Commission, 2021) recommend and certain national regulations (German Ordinance on the Protection of Animals and the Keeping of Farm Animals, 2006) stipulate that pullets must be reared in housing systems that correspond to those in which they will later be kept for egg production. However, there are no specific legal regulations in Europe for pullet rearing, although efforts are being made to establish such regulations. A key point of discussion is the timing of opening the aviary levels, at which point the chicks have access to litter after a certain initial rearing phase on the perforated grids of the aviary levels. During the first weeks of life, chicks in practical pullet rearing are confined to the aviary levels and are therefore not provided with access to litter on the barn floor in order to enable close animal observation, ensure safe access to feed and water, and make use of the temperature gradient within the height of the barn (Pottgüter et al., 2018). It is a common practice to open the aviaries at around 4 to 5 weeks of age (de Haas et al., 2014; Liebers et al., 2019) and practical recommendations suggest an age of 3 to 5 weeks, depending on the type of aviary (Pottgüter et al., 2018). However, the importance of the opening time of the aviaries for the occurrence of behavioural disorders has not yet been scientifically clarified. It is evident that rearing on litter during the first few weeks causes less feather pecking during the rearing period and later in lay than rearing on grids in pullets (reviewed by Janczak and Riber, 2015). On the other hand, the preventive effect against feather pecking by providing manipulable substrates such as alfalfa or chick feed in closed aviary levels has also been proven (de Haas et al., 2014; Tahamtani et al., 2016; Schreiter et al., 2020).

In addition to housing, nutrition is considered a key factor influencing feather pecking behaviour. Studies in adult laying hens have shown that an increased fiber intake is associated with a reduction in feather pecking and cannibalism, resulting in fewer pecking-related skin and toe injuries (Hartini et al., 2002; van Krimpen et al., 2005; Qaisrani et al., 2013). In this context, diets enriched with crude fiber may increase both the duration of feed intake and the overall feed intake (Hartini et al., 2002; van Krimpen et al., 2005). It is generally assumed that extending the feed intake period can satisfy the motivation to peck more effectively, thereby reducing the risk of pecking being redirected towards conspecifics (Kjaer and Bessei, 2013; Mens et al., 2020). Moreover, fiber-rich diets can stimulate the development of the gizzard and the gastrointestinal tract, thereby improving nutrient digestibility and feed intake capacity (Svihus et al., 2011; Liermann et al., 2019). A recent review recommends investigating the effects of fiber-rich feed on the prevention of feather pecking, including during rearing (Mens et al., 2020).

Keel bone damages are important animal welfare issues in laying hens with multifactorial causes (Rufener and Makagon, 2020; Thøfner et al., 2021). Keel bone damage is generally used as an umbrella term including both keel bone fractures and keel bone deviations (**KBD**) (Rufener and Makagon, 2020). Keel bone fractures are characterized by sharp bends, callus formation, or fracture lines resulting from traumatic impacts or excessive mechanical load, whereas keel bone deviations describe abnormal changes from the straight keel bone axis that are typically considered to develop more gradually due to chronic mechanical pressure or deformation processes (Rufener and Makagon, 2020; Thøfner et al., 2021). Although clinically relevant keel bone damage mainly occurs during the laying period, early developmental processes and mechanical influences during rearing may already contribute to the development of later keel bone alterations (Casey-Trott et al., 2017; Rufener and Makagon, 2020; Toscano et al., 2020). There is evidence that rearing pullets in complex systems that encourage movement promotes their locomotor development and reduces subsequent KBD by enabling them to land more safely on perches (Casey-Trott et al., 2017). The extent to which promoting mobility by opening aviary levels earlier affects the condition of the keel bone in pullets is unclear. In addition to improving locomotor skills and spatial navigation, increased exercise and use of elevated structures during rearing may positively influence bone development and bone strength through mechanical loading processes (Regmi et al., 2016; Casey-Trott et al., 2017). In addition to potential effects of housing and locomotor activity on keel bone health, associations between nutrition and keel bone health in pullets and laying hens are generally recognized (Rufener and Makagon, 2020; Toscano et al., 2020). Therefore, it appears potentially plausible that fibre-rich diets could indirectly influence skeletal development through effects on gizzard development and nutrient utilization (Svihus et al., 2011; Liermann et al., 2019), although this has not yet been investigated in pullets.

Therefore, the aim of this study was to investigate the effects of the time of opening the aviary and the dietary fiber contents on the condition of the plumage, skin and keel bone of pullets throughout the rearing period in a longitudinal design. It was hypothesized that an early aviary opening (i.e., early access to litter) and higher dietary fiber contents can reduce the occurrence of PD, SI and KBD during rearing.

## Materials and methods

In this study, we compared the integument characteristics of 3,872 pullets reared in an aviary, examining the effects of varying dietary fiber contents in their feed and different opening times of the aviary blocks. The study was carried out between January 2024 and May 2024 at the Bavarian State Estate, Experimental and Educational Center in Poultry, located in Kitzingen, Germany.

### Ethical statement

The animals were housed in compliance with the applicable legal standards of the European Union (Council Directive 1999/74/EC on the minimum standards for the protection of laying hens, 1999) as well as German national regulations (German Order on the Protection of Animals and the Keeping of Production Animals, 2006). The study design was reviewed by the Animal Welfare Officer of the Bavarian State Research Center for Agriculture and was not classified as an animal experiment.

### Animals, housing and management

For this study, one-day-old, non-beak-trimmed chicks of the brown-egg layer hybrid strain Lohmann Brown Classic (LB, Lohmann Breeders, Cuxhaven, Germany) were purchased from the hatchery of Lohmann Deutschland GmbH & Co. KG (Ankum, Germany) and housed in a rearing stable in 16 compartments. The rearing period lasted from the first to the 17th week of age.

**Fig. 1:**
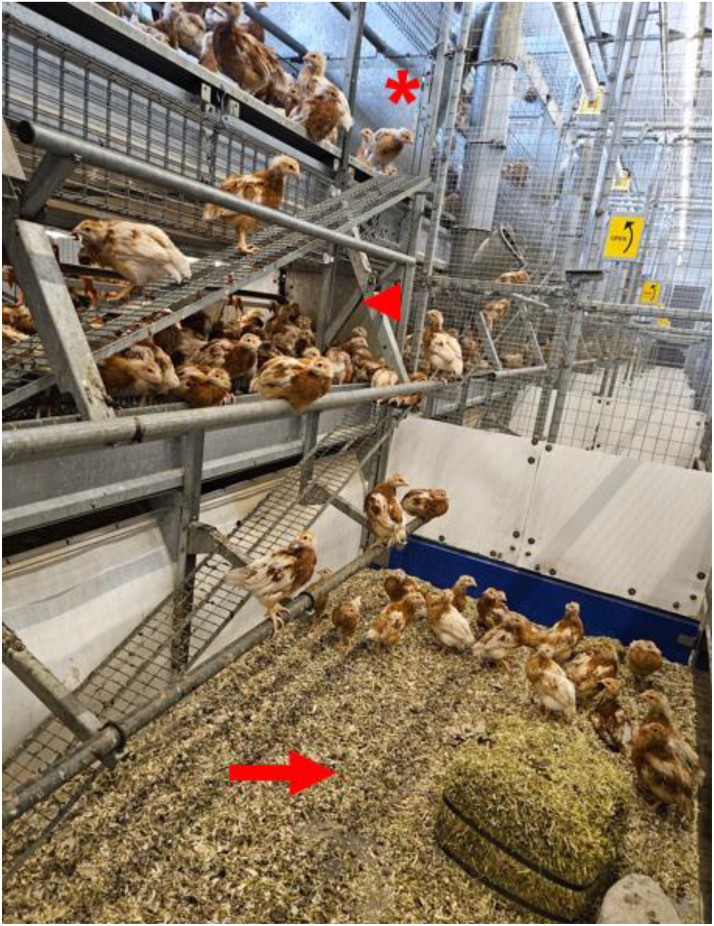
Compartment of the rearing facility with opened aviary levels. In addition to the fiber content, the opening times of the aviaries differed between the study groups: in 8 out of 16 compartments, the lower aviary level (arrow head) was opened on day 14, and the upper level (asterisk) on day 17 (group M14). In the remaining compartments, the lower aviary level was opened on day 28, and the upper level on day 31 (group M28). From the time the aviary was opened, the pullets had access to the littered floor area (arrow). Same area of littered floor area was available at the other side of each compartment.

**Fig. 2:**
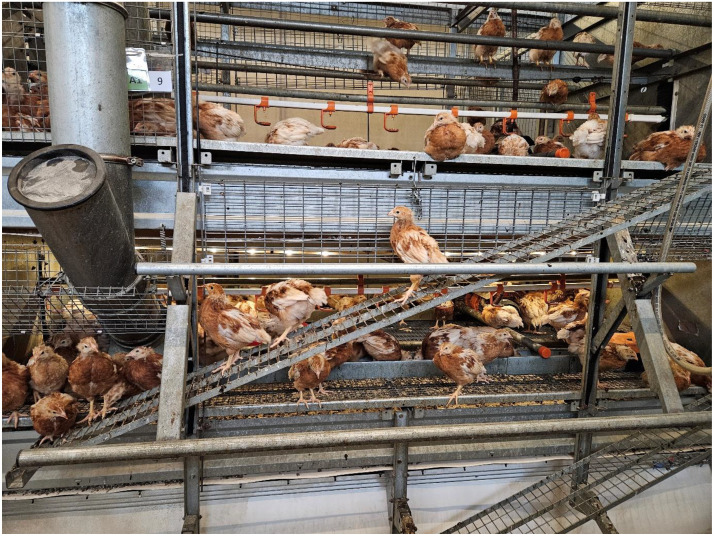
Aviary rearing facility used in the study with lower and upper aviary levels in the opened state, including perforated floor areas, a feed chain for feed supply, nipple drinkers for water supply, perches as elevated resting areas, and ramps as an additional option for moving between levels. Different indices between variants within an animal age indicate significant differences (p ≤ 0.05); Scoring protocol see Table 2; three-level scoring system based on a traffic light scheme with intact condition (score 0; green), mild alterations (score 1; yellow/orange), and severe alterations (score 2; red). The percentage given for the back/belly region per plumage assessment corresponds to the arithmetic mean of the two assessed plumage areas.

The rearing period was conducted in a facility equipped with a two-tiered aviary system (Natura Filia, Big Dutchman AG, Vechta-Calveslage, Germany), comprising 16 structurally identical compartments (each 12.8 m² in total area, consisting of 6.6 m² of slatted flooring and 6.2 m² of littered surface, including perches, feeding space and nipple drinkers). On the first day of life, 242 chicks per compartment were placed in the lower aviary level and on day 14, 121 chicks were transferred to the upper aviary level. Both aviary levels had equal floor areas and were equally equipped. Until the aviary blocks were opened (Fig. 1, Fig. 2), the chicks only had access to the perforated area in the aviary. Based on the total usable space offered in the aviary compartments (perforated plus littered area), the stocking density was 18.9 animals/m^2^.

To support feed intake during the first four weeks, 80% of the perforated area was initially covered with chick paper (corrugated roll board, REKA Wellpappenwerke, Kitzingen, Germany). From day 1 to 8, chick starter feed (Table 1) was distributed on this paper. The floor of the stable area was covered with softwood shavings (Premiumspan®, Hobelspanverarbeitung GmbH, Dittersdorf, Germany) and straw pellets (straw pellet, Einstreuprofi, Seelingstädt, Germany).

**Table 1:** Ingredients of the complete diets for pullets (% as is) used in the study: identical starter feed and two variants (Fcon and F+) in the grower and developer phases.

Ingredient	Starter (0 – 3 week)	Grower (4 – 8 week)		Developer (9 – 17 week)			
				Batch 1 (9 – 12 week)		Batch 2 (13 – 17 week)	
		Fcon^1^	F + 2	Fcon	F+	Fcon	F+
Wheat	39.2	41.8	29.4	42.1	28.7	45.5	32.1
Soybean meal, 48% CP	22.4	9.50	0.47	3.70	-	4.23	-
Corn	15.0	15.0	15.0	15.0	15.0	15.0	15.0
Wheat (high protein)	5.00	5.00	5.00	5.00	5.00	5.00	5.00
Rapeseed expeller	4.00	8.00	15.0	10.0	12.5	10.0	13.3
Sunflower meal (high protein)	4.00	8.00	15.0	10.0	15.0	10.0	15.0
Soybean oil	2.00	1.00	3.00	1.00	4.28	1.00	4.15
Peas	2.00	4.00	6.00	4.00	4.00	-	-
Limestone, fine	1.67	1.78	1.84	1.64	1.69	1.65	1.68
Wheat bran	1.25	3.00	6.00	5.00	9.00	5.00	9.00
Monocalcium phosphate	1.26	0.65	0.20	0.28	-	0.27	-
L-Lysine sulfate	0.34	0.42	0.52	0.31	0.35	0.35	0.40
Feed acid	0.30	0.30	0.30	0.30	0.30	0.30	0.30
Premix^3^	0.30	0.30	0.30	0.30	0.30	0.30	0.30
Lignocellulose^4^	0.25	0.25	0.73	0.25	2.58	0.25	2.41
Alfalfa meal	0.25	0.25	0.50	0.50	0.75	0.50	0.75
DL-Methionine	0.25	0.20	0.17	0.10	0.08	0.09	0.07
Sodium chloride	0.23	0.21	0.23	0.18	0.18	0.18	0.18
Sodium bicarbonate	0.23	0.30	0.25	0.34	0.33	0.34	0.33
L-Threonine	0.08	0.07	0.07	0.02	0.02	0.02	0.02
L-Isoleucine	-	0.02	0.04	-	-	-	-

1 Fcon: standard fiber content.

2 F+: moderate elevated fiber content.

3 Premix provided per kg diet: 10,000 IU vitamin A (3a672a); 3,200 IU vitamin D_3_ (3a671); 50.0 mg vitamin E (3a700i); 15.0 mg copper (copper(II) sulphate pentahydrate) (3b405); 50.0 mg iron (iron(II)-carbonate) (3b101); 100 mg manganese (manganese II oxide) (3b502); 60.0 mg zinc (zinc sulfate monohydrate) (3b605); 1.00 mg iodine (calcium ioodate, anhydrous) (3b202); 0.20 mg selenium (sodium selenite) (3b801); 3050 - 5187 mg L-lysine sulfate (3c324); 2,040 mg formic acid (1k236); 600 mg propionic acid (1K280); 2,000 U endo-1,4-ß-Xylanase (EC 3.2.1.8) 4a11; 300 U endo-1,4 β-Xylanase (EC 3.2.1.8) 4a10; 4,000 U subtilisin (EC 3.4.21.62) 4a10; 400 U alpha- amylase (EC 3.2.1.1) 4a10; 1,250 VU endo-1,4-β-Xylanase (EC 3.2.1.8) 4a22; 860 VU endo-1,3(4)-β - glucanases (EC 3.2.1.6) 4a22; 500 FTU 6- Phytase (EC 3.1.3.26) 4a37.

4 OptiCell®.

Each compartment contained ramps (Big Dutchman AG, Vechta-Calveslage, Germany) leading from the floor to the aviary levels (one ramp per aviary level and side, Fig. 1, Fig. 2). These were in place from the time the aviary was opened until week 10.

Within the aviary system, drinking water was supplied via nipple drinkers (Big Dutchman AG, Vechta-Calveslage, Germany) and feed was supplied via a flat chain feeder (Big Dutchman AG, Vechta-Calveslage, Germany). Each pullet had 10 cm of a round metal bar (Big Dutchman AG, Vechta-Calveslage, Germany) available as a perch space, of which 6 cm were located within the aviary levels and 4 cm outside the aviary above the littered area. No elevated platforms were installed in the aviary system.

As additional enrichment material, the animals had ad libitum access to pecking stones (Vilolith medium, Deutsche Vilomix Tierernährung GmbH, Neuenkirchen-Vörden, Germany) and alfalfa blocks (Einstreuprofi, Seelingstädt, Germany) from the first day of life. These enrichment materials were offered in the aviary until the 10th day after the aviaries were opened, as well as on the floor of the barn on the day the aviaries were opened. Once a week, the animals were given grit (casafino Filtersand, BayWa AG, Munich, Germany) in grain sizes of 0,7-1,2 mm (1st–2nd week of age, 1.5 g/week) or 2,0–3,2 mm (from 3rd week of age, 1.5 g/week).

Light management followed a step-down regime based on established management recommendations (Lohmann Breeders, 2021). A vaccination protocol appropriate to the flock’s use, location, and health status was followed throughout the rearing period in accordance with established guidelines (Lohmann Breeders, 2021) to promote flock health and minimize mortality in the later production phase.

### Study design, and data collection

A 2 × 2 factorial study design was used. Firstly, the aviary's opening days were varied and secondly, two different feeding strategies with varying fiber contents were used. The observation period was from the day the pullets were placed in the house until week 17.

In 8 out of 16 compartments, access to the littered barn floor was given by opening the lower level of the aviary on day 14 and the upper level on day 17 (**M14**). In the remaining compartments (**M28**), the lower and upper aviary levels were opened later, on days 28 and 31, respectively (Fig. 1). Opening the aviary levels at slightly different times is in line with practical recommendations (Pottgüter et al., 2018) to ensure more targeted observation in the first few days of the open aviary.

Regarding the feeding strategy, two study groups were established, each consisting of eight compartments: a control group (**Fcon**) with a fiber content typically used under practical rearing conditions, and a treatment group (**F+**) receiving a diet with increased fiber content. The composition and nutrient contents of the two feed types for the grower (weeks 4–8) and developer (weeks 9–17) phases are presented in Table 1, Table 2. During the first three weeks, all animals received an identical chick starter feed (Table 1, Table 2). Except for crude fiber and neutral detergent fiber (aNDFom) content, the Fcon and F+ diets were formulated to be isonitrogenous and isoenergetic. All feed was supplied to the research station as ready-to-use compound feed from BEST 3 Geflügelernährung GmbH (Twistringen, Germany).

**Table 2:** Calculated and analyzed nutrient (% as is, unless otherwise stated) and calculated energy (MJ AME_N_/kg as-fed) content of the complete diets for pullets used in this study: identical starter feed and two variants (Fcon and F+) in the grower and developer phases.

	Starter (0 – 3 week)	Grower (4 – 8 week)		Developer (9 – 17 week)			
				Batch 1 (9 – 12 week)		Batch 2 (13 – 17 week)	
		Fcon^1^	F + 2	Fcon	F+	Fcon	F+
**Calculated composition**							
Dry matter	88.1	88.0	88.6	88.0	88.7	88.2	88.9
Crude protein	20.0	17.5	17.5	16.3	16.3	16.3	16.3
Ether extract	4.93	4.22	6.73	4.39	7.76	4.34	7.68
Starch	38.7	41.3	35.3	41.7	34.4	42.1	34.7
Sugar	4.18	3.77	3.87	3.64	3.73	3.61	3.71
Crude fiber	4.00	5.18	7.56	5.84	8.54	5.56	8.20
Crude ash	6.18	5.72	5.82	5.31	5.44	5.34	5.46
aNDFom^3^	13.5	16.1	19.4	17.4	21.0	17.1	20.6
ADFom^4^	5.8	7.4	10.4	8.3	11.3	7.9	10.9
Calcium	1.00	0.95	0.95	0.85	0.85	0.85	0.85
Phosphorus	0.70	0.60	0.60	0.55	0.55	0.55	0.56
Sodium	0.16	0.17	0.17	0.17	0.17	0.17	0.17
Lysine	1.14	0.98	1.00	0.83	0.84	0.82	0.83
Methionine	0.55	0.48	0.48	0.38	0.38	0.37	0.38
AME_N_ (MJ/kg)	11.8	11.5	11.4	11.5	11.4	11.5	11.4
**Analyzed composition**							
Dry matter	88.0	88.0	88.0	88.0	88.0	88.0	88.0
Crude protein	20.3	16.7	15.6	15.3	15.6	16.3	15.5
Ether extract	4.60	3.60	5.40	3.80	6.60	3.20	7.10
Starch	38.7	45.6	42.4	43.6	36.0	43.5	36.5
Sugar	4.00	3.40	3.40	3.20	3.50	3.20	3.40
Crude fiber	4.40	4.90	6.80	5.80	7.50	5.70	8.10
Crude ash	6.00	4.30	4.00	4.80	5.80	5.20	5.00
aNDFom^3^	11.0	12.3	15.5	15.0	17.2	14.6	19.0
ADFom^4^	6.50	6.90	10.6	8.10	9.60	8.90	13.6
Calcium	1.01	0.61	0.55	0.79	1.03	0.89	0.83
Phosphorus	0.79	0.61	0.61	0.60	0.60	0.58	0.57
Sodium	0.18	0.12	0.09	0.16	0.26	0.18	0.16
Lysine	1.23	0.97	0.81	0.74	0.78	0.85	0.84
Methionine	0.92	0.45	0.48	0.35	0.33	0.77	0.38
AME_N_ (MJ/kg)	11.7	11.9	11.8	11.4	11.2	11.3	11.4

1 Fcon: standard fiber content.

2 F+: moderate elevated fiber content.

3 Amylase-treated neutral detergent fiber with ash correction.

4 Acid detergent fiber with ash correction.

Combining the two factors investigated in this study (i.e., aviary opening times and feeding groups) resulted in the following four variants: i) aviary opening at 28 and 31 days and diet with standard fiber content (**M28_Fcon**), ii) aviary opening at 28 and 31 days and diet with moderate elevated fiber content (**M28_F+**), iii) aviary opening at 14 and 17 days and diet with standard fiber content (**M14_Fcon**), and iv) aviary opening at 14 and 17 days and diet with moderate elevated fiber content (**M14_F+**).

To indirectly quantify severe feather pecking and cannibalism, an assessment of the integument was carried out (Schwarzer et al., 2022). The calculation of the necessary sample size was based on Schreiter et al., 2020. In this context, individual birds served as observational units. Sample size determination was based on a power analysis with preliminary data (not shown, calculations were carried out using: https://imsiewebarchiv.uni-koeln.de/beratung/rechner/ps.html). To detect a 15% difference in the proportion of birds with integument damage with a power of 0.80 and a significance level of α = 0.05, at least 113 birds per treatment group were required.

During the rearing period, evaluations were conducted in weeks 4, 8, 12, and 16. At each time point, 30 pullets per compartment were assessed, corresponding to approximately 12.4% of the animals within each compartment (242 birds per compartment). The sample size was determined based on an a priori sample size calculation, as described below. The assessed individuals comprised birds from the lower aviary level (n = 7), upper aviary level (n = 7), middle scratching area (n = 8), and outer scratching area (n = 8). At each assessment time point, 120 hens were assessed for each of the four treatment groups. The integument and KBD were scored by three observers who had completed intensive training on 500 animals and also performed an inter-observer comparison on 60 animals to determine inter-observer reliability (see statistical analyses for calculation).

The assessment of integument condition followed the protocol described by Keppler, 2017, encompassing the evaluation of plumage condition and SI (Table 3). Plumage scoring was conducted separately for the back, belly (including the cloacal region and ventral rump), and wing feathers. The individual scores for the plumage of the back and the plumage of the belly were summed in accordance with Schreiter et al., 2020 and combined into a back/belly plumage score. Feather coverage on the front of the neck and the breast were excluded from the assessment, as damage in these areas is often due to mechanical abrasion from the feeder and is not a reliable indicator for severe feather pecking (Bilcik and Keeling, 1999). Regrowing feathers were classified as present feathers and were therefore not considered plumage damage within the scoring procedure in order to minimize potential confounding effects of physiological moulting during rearing. In the evaluation of skin and feather follicle injuries in pullets, all body areas – excluding the head and feet including toes – were considered.

**Table 3:** Scoring system for visual assessment of plumage condition and skin injuries, and for keel bone palpation.

Trait	Score 0	Score 1	Score 2
Plumage damage (back plumage/belly plumage)^1^	intact plumage: complete plumage, no damage of single feathers	moderate plumage damage: ≥ 1 featherless areas with a maximum diameter of 1 cm each, damaged feathers present (deformed or broken)	severe plumage damage: ≥ 1 featherless areas with a diameter > 1 cm
Wing feather loss^1^	intact plumage: complete plumage, no damage of single feathers	moderate plumage damage < 3 damaged feathers (missing corners in feather vanes)	severe plumage damage ≥ 3 damaged feathers (missing corners in feather vanes)
Skin injuries^1^	intact skin: no injuries on skin or injured blood-filled feather follicles	moderate injuries: injured blood-filled feather follicles	severe injuries: one or more injuries/wounds of the skin characterized by visible disruption of skin integrity
Keel bone deviation^2^	no deviation: no deviation from the straight midline of the keel bone	mild deviation: ≤ 0.5 cm deviation from straight midline of the keel bone	severe deviation: > 0.5 cm deviation from straight midline of the keel bone

1 : scheme according to Keppler, 2017,.

2 : modified scheme according to Keppler, 2017 and Jung et al., 2020.

For all traits, a three-level scale (Keppler, 2017) was applied, with a score of 0 representing an intact condition, a score of 1 representing moderate damage, and a score of 2 representing severe damage. The keel bone was examined by palpation for KBD, following a modified protocol (Table 3) based on Keppler, 2017 and Jung et al., 2020.

### Statistical analyses

Microsoft Excel (Version 2021, Microsoft Corporation, Redmond, WA, USA) was used for data entry, preprocessing, and generating selected graphs. All subsequent descriptive and inferential statistical evaluations were performed using IBM SPSS Statistics (Version 31, SPSS Inc., Chicago, IL, USA).

A concordance analysis was performed to assess the agreement in the scoring of the integument and the KBD. The prevalence-adjusted and bias-adjusted Kappa coefficient (PABAK) was used to quantify interobserver reliability, following Gunnarsson et al., 2000. The interpretation of PABAK values was based on the scale provided by Landis and Koch, 1977 and Kwiecien et al., 2011: ≤0.20 = poor, 0.21–0.40 = fair, 0.41–0.60 = moderate, 0.61–0.80 = good, and >0.80 = very good agreement.

In a first step, the ordinally scaled integument and KBD characteristics were analyzed univariately for differences between the treatment group, using the Kruskal–Wallis test (du Prel et al., 2010). Where applicable, post hoc pairwise comparisons between the four experimental variants were conducted using the Mann–Whitney–U test, following identification of significant main effects (du Prel et al., 2010).

In a second step, we performed multivariable analyses as interference statistics. Binary logistic regression (BLR) models were applied to analyze integument and keel bone traits, following the approach described by Baltes-Götz, 2000. Multiple logistic regression, rather than ordinal regression, was chosen because some score categories contained very few observations and the proportional odds assumption was not met in all cases (McCullagh, 1980). To increase statistical power and facilitate the identification of predictors, integument and KBD scores were dichotomized. Specifically, ordinal scaling (as defined by Keppler, 2017; Jung et al., 2020) was converted into nominal scaling, with a score of 0 assigned to observations with a score of 0, and a score of 1 assigned to observations with scores ≥1. In the BLR model, the PD, wing feather loss, SI and KBD were used as dependent variables. Opening time of the aviary, feeding, age and the interaction opening time of the aviary*feeding were included in the models as explanatory variables. Compartment was included within the BLR model structure to account for potential non-independence and clustering of animals within the same pen. The absence of multicollinearity was ensured by calculating the Pearson’s correlation coefficient and performing a collinearity diagnosis with the variance inflation factor and condition index (Menard, 1995; Field, 2013).

Across all inferential analyses, statistical significance was defined at P ≤ 0.05.

## Results and discussion

The agreement in scoring by multiple observers of the integument and keel bone characteristics, as central features of the study, was quantified by a concordance analysis. The results for inter-observer reliability in integument and keel bone assessment are shown in Table 4. For PD, wing feather loss and SI, high PABAK values in the range of 0.84–0.98 were found, which, according to Landis and Koch, 1977, indicate very good agreement in scoring. For palpation of the keel bone, lower PABAK values were obtained with a median of 0.73 for the observer combinations, which indicates good agreement in scoring (Landis and Koch, 1977). Jung et al., 2020 achieved a slightly lower PABAK value of 0.8 in their study, with scoring by several observers, for PD, and a slightly higher PABAK value of 0.8 for KBD.

**Table 4:** Prevalence-adjusted and bias-adjusted kappa (PABAK) as a measure of inter-observer reliability. Three observers (O1, O2 and O3) assessed the condition of 60 pullets with plumage damage in two areas of the body (back and belly), wing feather loss, skin injuries and keel bone deviations.

Observer combination	Plumage damage (n = 120)		Wing feather loss (n = 60)		Skin injuries (n = 60)		Keel bone deviation (n = 60)	
	agreement (%)	PABAK	agreement (%)	PABAK	agreement (%)	PABAK	agreement (%)	PABAK
O1/O2	89.2	0.84	98.3	0.98	90.0	0.85	81.7	0.73
O1/O3	96.7	0.95	98.3	0.98	95.0	0.93	81.7	0.73
O2/O3	90.8	0.86	96.7	0.95	95.0	0.93	73.3	0.60
*Median*	90.8	0.86	98.3	0.95	95.0	0.93	81.7	0.73

**Fig. 3:**
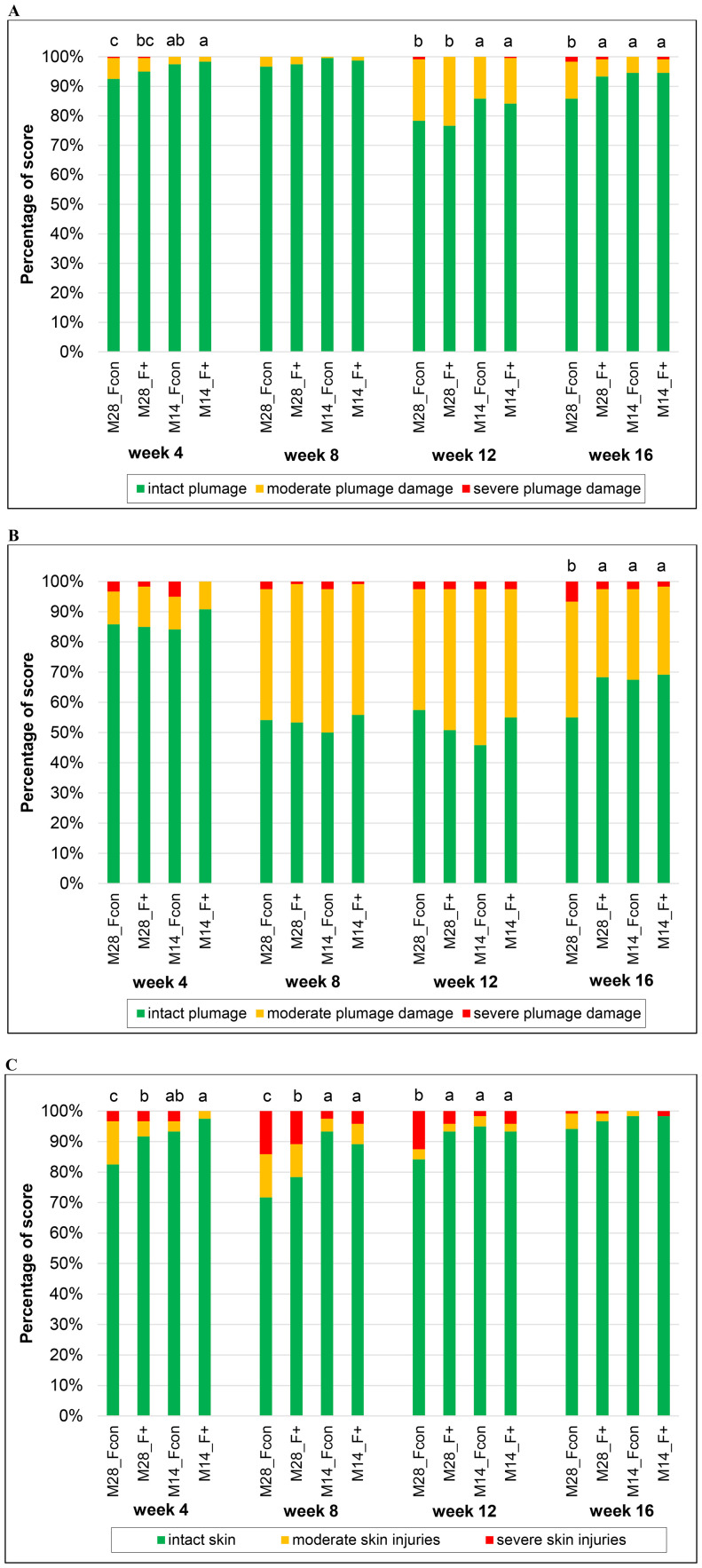
Plumage damage on back/belly (A), wing feather loss (B), and skin injuries (C) in pullets of four study groups at different ages. Integument characteristics are shown for pullets with aviary opening on day 28 and 31 receiving either standard fiber content (M28_Fcon) or moderate elevated fiber feed (M28_F+), and for pullets with aviary opening on day 14 and 17 receiving either standard fiber content (M14_Fcon) or moderate elevated fiber feed (M14_F+). Scoring protocol see Table 2; three-level scoring system based on a traffic light scheme with intact condition (score 0; green), mild alterations (score 1; yellow/orange), and severe alterations (score 2; red).

**Fig. 4:**
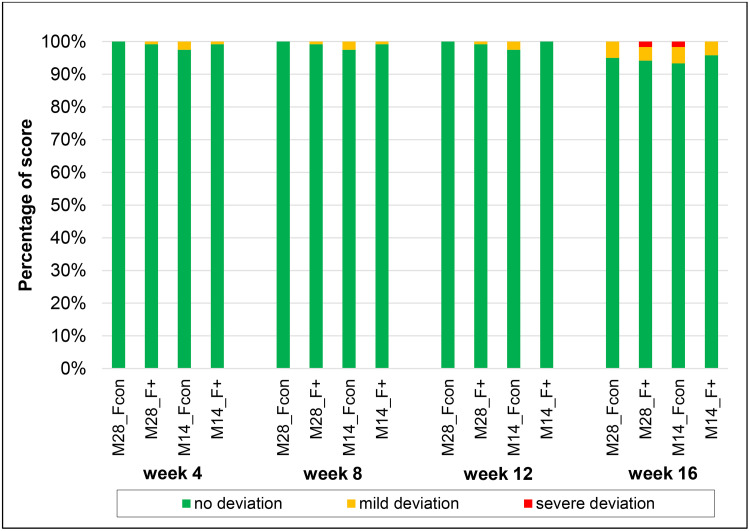
Keel bone deviations in pullets of four study groups at different ages. Keel bone characteristics are shown for pullets with aviary opening on day 28 and 31 receiving either standard fiber content (M28_Fcon) or moderate elevated fiber feed (M28_F+), and for pullets with aviary opening on day 14 and 17 receiving either standard fiber content (M14_Fcon) or moderate elevated fiber feed (M14_F+).

The results of the measurements of the PD, wing feather loss, and SI are shown in Fig. 3, whereas KBD results are shown separately in Fig. 4. In the back/belly plumage, the proportion of intact plumage is significantly higher at 4 weeks (95.8%) and 8 weeks (98.1%), than at 12 weeks (81.3%). The proportion of undamaged plumage increased again to 92.1% at week 16. The improvement in plumage condition can be explained by intensive moulting processes during rearing which can typically found in week 12 to 14 (Pottgüter et al., 2018). In contrast, a fundamentally irreversible increase in plumage damage can be observed at flock level during a laying period (Spindler et al., 2016; Schreiter and Freick, 2023). Numerically, more plumage damage was observed on the wings, with the proportion of intact plumage also being lowest in week 12. An effect of the experimental variant was found for the PD as a result of the univariate analysis in weeks 4, 12, and 16 (P ≤ 0.025). In week 4, M28_Fcon (7.1% moderate and 0.4% severe alterations) showed the highest occurrence of PD, and M14_F+ the lowest (1.7% moderate alterations). M14_Fcon (2.5% moderate alterations) was indifferent to M14_F+ and M28_F+ (4.6% moderate and 0.4% severe alterations), but had better plumage condition than M28_Fcon. M28_F+ differed in its PD only from M14_F+. In week 12, a higher occurrence of PD was found in both feed variants with aviaries opened on day 28 (M28_Fcon: 20.8% moderate and 0.8% severe alterations; M28_F+: 23.3% moderate and 0.0% severe alterations), than in both feed variants with aviaries opened on day 14 (M14_Fcon: 14.2% moderate alterations; M14_F+: 15.4% moderate and 0.4% severe alterations). The effect of the variant was then limited, in week 16 to the difference between M28_Fcon (12.5% moderate and 1.7% severe alterations) and the three other variants (4.6-5.8% moderate and 0.0-0.8% severe alterations). In terms of wing feather loss, the treatment group had an effect only at week 16 (P = 0.042), with the same directional effect and identical ranking as for back/belly feathers at this age. M28_Fcon showed more feather damage (38.3% moderate and 6.7% severe alterations) than the three other variants (29.2-30.0% moderate and 1.7-2.5% severe alterations).

Moderate or severe SI, including lesions of the blood-filled feather follicles, were observed in 8.8% of the 4-week-old pullets, 16.9% of the 8-week-old pullets, 8.5% of the 12-week-old pullets, and 3.1% of the 16-week-old pullets. The treatment group had an effect on the occurrence of SI in weeks 4, 8 and 12 (P ≤ 0.008), but not in week 16 (P = 0.210). One explanation for the varying effects at different ages may be the length of daylight, which is typically adjusted according to age in the rearing of pullets and, in our study, had a constant daylight length of 9 hours after the light step-down from week 9 onwards. In week 4, the proportion of intact skin was highest in M14_F+ (97.5%), and lowest in M28_Fcon (82.5%). M28_F+ (91.7% intact skin) differed from both of the above variants; M14_Fcon (93.3% intact skin) differed only from M28_Fcon. The ranking for week 8 was similar, with M28_Fcon having the lowest (71.7%), M28_F+ having intermediate (78.3%) and M14_Fcon (93.3%) and M14_F+ (89.2%) having the highest proportion of intact skin. At the next assessment, in week 12, there was a difference between M28_Fcon (84.2% intact skin) and the three other variants (93.3-95.0% intact skin). When looking at the differences between the two feeding variants (Fcon vs. F+) within the respective management variant (M14 vs. M28), the following is noticeable: At all three time points with effects of the treatment group, M28_Fcon showed significantly more SI than M28_F+, but there were no differences within the two variants with aviary opening on day 14 (M14_Fcon vs. M14_F+).

There were no differences in KBD between the treatment groups (P ≥ 0.110). Keel bone deviations in week 4 (1.0%), 8 (1.0%), and 12 (0.8%) occurred only as slight deviations with very low occurrence. The very low occurrence of KBD in weeks 4 to 12 should be interpreted with caution, as only mild deviations were detected and no differences among treatment groups were observed. Because the keel bone is not yet fully ossified at this age, early and slight deviations from the straight midline may reflect individual developmental variation, localized mechanical pressure, or methodological uncertainty associated with palpation in very young birds. Therefore, no treatment-related effects should be inferred from these early findings. At week 16, in addition to 94.6% intact keel bones, 4.6% mild and 0.8% severe KBD were observed. The low occurrence of moderate to severe KBD in 16-week-old pullets (5.4%) is consistent with the observations of Casey-Trott et al., 2017, who also reported only a very small proportion of affected animals at this age. In their study, Habig et al., 2021 found that keel bone damage occurred in only 0.5 to 3.0% of 15-week-old pullets from various laying hybrid strains. In contrast, Emmert et al., 2024 reported significantly higher occurrence rates (24.3%) in 16-week-old pullets. One potential explanation for these variations is the methodology: While palpation was used in the studies by Casey-Trott, 2017 and Habig et al., 2021; Emmert et al., 2024 applied image-based quantitative computed tomography, which allows for more sensitive detection of subtle deformities. It has been shown that KBD and keel bone fractures occur more frequently in hens during the laying phase (Rufener and Makagon, 2020; Thøfner et al., 2021).

BLR models analyzed the associations of PD, wing feather loss, SI, and KBD with animal age, aviary opening times, feeding, and the interaction opening time of the aviary*feeding (Table 5). Animal age had an effect on all examined traits (P < 0.001). Neither the feeding nor the aviary opening time variant had any influence on wing feather loss or KBD (P ≥ 0.148). The back/belly plumage and skin of the pullets showed more damage with Fcon than with F+ (PD: OR = 0.65, P = 0.012; SI: OR = 0.53, P = 0.004) and less damage with M14 than with M28 (PD: OR = 0.36, P < 0.001; SI: OR = 0.19, P < 0.001). Thus, a higher fiber content in the feed and earlier opening of the aviaries were associated with less PD and SI.

**Table 5:** Effects of feeding, aviary opening time, age and the interaction feeding x aviary opening time on plumage damage, wing feather loss, skin injuries and keel bone deviations in brown layer pullets – results of binary logistic regression models.

Trait	Score 1 (%)	Coefficients (SE)	Odds ratio (95% CI)	p-value
**Plumage damage**				
*Feeding*				
Standard fiber content	14.5	Reference	Baseline	
Moderate elevated fiber content	10.9	−0.44 (0.17)	0.65 (0.46-0.87)	0.012
*Aviary opening time*				
Aviary opening on day 28	8.3	Reference	Baseline	
Aviary opening on day 14	17.1	−1.01 (0.20)	0.36 (0.25-0.54)	<0.001
*Age*		0.09 (0.02)	1.10 (1.07-1.14)	<0.001
*Feeding x Aviary opening time*		0.36 (0.29)	1.43 (0.80-2.55)	0.233
Intercept		−2.30 (0.25)		
**Wing feather loss**				
*Feeding*				
Standard fiber content	37.5	Reference	Baseline	
Moderate elevated fiber content	34.0	−0.05 (0.14)	0.95 (0.73-1.24)	0.685
*Aviary opening time*				
Aviary opening on day 28	35.2	Reference	Baseline	
Aviary opening on day 14	36.3	0.55 (0.13)	0.99 (0.76-1.29)	0.928
*Age*		0.07 (0.01)	1.08 (1.05-1.10)	<0.001
*Feeding x Aviary opening time*		−0.17 (0.12)	0.85 (0.58-1.24)	0.395
Intercept		−0.88 (0.17)		
**Skin injuries**				
*Feeding*				
Standard fiber content	10.3	Reference	Baseline	
Moderate elevated fiber content	7.7	−0.61 (0.19)	0.53 (0.36-0.79)	0.004
*Aviary opening time*				
Aviary opening on day 28	13.4	Reference	Baseline	
Aviary opening on day 14	4.6	−1.68 (0.27)	0.19 (0.11-0.32)	<0.001
*Age*		−0.07 (0.01)	0.93 (0.89-0.96)	<0.001
*Feeding x Aviary opening time*		1.01 (0.37)	2.74 (1.32-5.67)	0.009
Intercept		−0.78 (0.26)		
**Keel bone deviation**				
*Feeding*				
Standard fiber content	2.4	Reference	Baseline	
Moderate elevated fiber content	1.8	0.11 (0.47)	1.11 (0.45-2.78)	0.817
*Aviary opening time*				
Aviary opening on day 28	2.5	Reference	Baseline	
Aviary opening on day 14	1.7	0.61 (0.42)	1.85 (0.81-4.22)	0.148
*Age*		0.15 (0.04)	1.17 (1.08-1.26)	<0.001
*Feeding x Aviary opening time*		−0.86 (0.64)	0.42 (0.12-1.48)	0.180
Intercept		−5.42 (0.66)		

SE – standard error, CI – confidence interval; Score 0 — intact condition of the integument or keel bone, score 1 — changes in the condition of the integument or keel bone.

The only trait showing a significant interaction between feeding and aviary opening was SI (P = 0.009). This indicates that the effect of fiber content on skin condition differed between the two aviary opening variants. Considering all four variants, a reduction in SI with higher fiber content was evident only in pullets when aviaries were opened at 28 days. In contrast, no significant differences were observed between M14_Fcon and M14_F+ in the univariate analysis (Fig. 3). One possible explanation could be that the earlier access to litter provided additional opportunities for exploratory pecking and oral manipulation, which may have partially compensated for the lower dietary fiber content in the Fcon groups. However, the actual intake of litter material was not quantified in the present study. Since early access to litter generally has a preventive effect against feather pecking and cannibalism (reviewed by Janczak and Riber, 2015), it still seems possible that the earlier opening of the aviary fundamentally reduced the basal risk of behavioral disorders, to such an extent that the feed effect was no longer detectable. The observed interaction suggests that fiber-rich feed can only develop its full preventive potential when alternative foraging activities – such as litter – are not (yet) available. Further studies should specifically investigate the additive and potentially synergistic effects of both factors.

The results on the positive effect of a higher fiber content on plumage condition are consistent with studies in adult laying hens, which have shown that increased fiber content in feed can stimulate foraging behavior and reduce feather pecking, through prolonged feed intake and longer satiety (Hartini et al., 2002; van Krimpen et al., 2005; Kjaer and Bessei, 2013; Qaisrani et al., 2013; Mens et al., 2020). Fiber-rich feed mixtures also improve digestive health and promote gizzard development (Svihus et al., 2011; Liermann et al., 2019), which has been shown to contribute to animal health (Mens et al., 2020). In the present trial, this may have favored better integument quality in the F+ group, especially in environments with limited activity opportunities due to later access to litter.

Since an earlier aviary opening was associated with a significant improvement in plumage and skin condition, it can be assumed that this measure reduced the risk of feather pecking and cannibalism. Early access to structured substrates such as litter is linked to a reduction in feather pecking and cannibalism, as it encourages the animals to engage in species-specific scratching and exploration behavior (reviewed by Janczak and Riber, 2015). However, studies enrolled in the review by Janczak and Riber, 2015 mainly refer to systems with extremely limited environmental stimuli, whereas modern aviary systems often already provide enrichment materials and partially paper-covered slatted floors on the aviary levels. The latter two measures have been shown to reduce feather damage in more recent studies (de Haas et al., 2014; Tahamtani et al., 2016; Schreiter et al., 2020). Against this background, it seems plausible that the timing of aviary opening (i.e., unrestricted access to litter) is only one aspect of the complex interaction between environment, behavior and animal health. In particular, it is important to examine how management measures for early aviary opening, e.g. to prevent pullets from spending the night in the litter area, can be implemented in large, commercially used rearing aviary houses. Field studies appear appropriate for this purpose.

The fact that the main effects examined in the study had similar effects on PD and SI underscores that both behavioral disorders have very similar causes and that areas of skin exposed as a result of feather pecking promote the occurrence of cannibalism (Spindler et al., 2016; van Staaveren et al., 2021; Schwarzer et al., 2022).

The lack of effect of the aviary opening time on KBD should be considered in particular in light of the expected low occurrence of palpable KBD in pullets. Nevertheless, recent studies indicate that keel bone ossification proceeds gradually and with considerable individual variation during rearing, while slight deviations may already develop before complete ossification is achieved (Gretarsson et al., 2024). Several reviews on keel bone damage in laying hens have therefore emphasized the importance of investigating keel bone alterations already during the pullet rearing period. Although the palpation-based approach used in the present study can only contribute partially to this research field, the obtained findings may represent important building blocks for a better understanding of the early development of keel bone alterations. In addition, early access to complex housing environments could promote locomotor development and potentially reduce the subsequent risk of keel bone damage during the laying period. The effects of rearing on keel bone health should therefore be monitored further during the laying period.

In addition to the limitations already discussed regarding the generalizability and interpretation of the findings, several further aspects should be considered when interpreting the results of the present study.

With regard to the general sampling procedure used for the assessed birds in this study, it should be considered that individual birds may differ in their use of specific areas within the aviary system, which could potentially influence both integument condition and keel bone characteristics. At the same time, the location of a bird at the time of assessment does not necessarily reflect its long-term spatial use behaviour within the system.

Furthermore, physiological moulting processes during the rearing period may have influenced the assessment of plumage condition. Although the applied scoring system was specifically developed for chicks and pullets and regrowing feathers were generally classified as present feathers, there is potentially a very small possibility that plumage scoring was minimally influenced by moulting processes.

The present study focused exclusively on the rearing period and therefore does not yet allow conclusions regarding whether the observed effects persist during the subsequent laying period.

## Conclusion

This study provides new insights into how management measures and feeding strategies during rearing influence the integument condition of pullets. When considering the two main effects examined, it can generally be concluded that increasing the fiber content and opening the rearing aviaries earlier can improve the plumage and skin condition as indirect indicators of severe feather pecking and cannibalism during the rearing period. The handling of chicks with access to litter from day 14 in commercial aviary systems under production conditions still requires further evaluation.

Notably, an interaction between feeding and aviary opening was observed: a positive effect of fiber on skin integrity occurred only with later litter access. The mechanisms underlying this interaction warrant further investigation. Overall, our findings highlight the importance of targeted housing and feeding strategies during pullet rearing to prevent integument damage and promote animal welfare.

Since the present study specifically focused on the rearing period, the findings should primarily be interpreted within this developmental phase. Further studies are required to investigate whether the observed effects persist during the subsequent laying period and to evaluate possible long-term implications for animal welfare and production performance.

## Declaration of AI and AI-assisted technologies in the writing process

In the preparation of this manuscript, the authors used DeepL and ChatGPT (OpenAI) to support translation, grammar correction, and linguistic refinement. All content was subsequently reviewed and revised by the authors, who take full responsibility for the final version of the manuscript.

## CRediT authorship contribution statement

**R. Schreiter:** Conceptualization, Methodology, Investigation, Formal analysis, Data curation, Visualization, Writing – original draft, Writing – review & editing. **L. Fitz:** Investigation, Data curation, Writing – review & editing. **J. Krieg:** Methodology, Formal analysis, Writing – review & editing. **P. Schulte-Huxel:** Methodology, Validation, Writing – review & editing. **A. Schröder:** Investigation, Methodology, Data curation, Writing – review & editing. **J. Slama:** Methodology, Investigation, Data curation, Writing – review & editing. **P. Hofmann:** Conceptualization, Methodology, Resources, Supervision, Funding acquisition, Writing – review & editing. **H. Louton:** Conceptualization, Methodology, Resources, Supervision, Writing – review & editing.

## Disclosures

The authors have no conflicts of interest to declare.
